# Ocular Surface Displacement with and without Contact Lenses during Non-Contact Tonometry

**DOI:** 10.1371/journal.pone.0096066

**Published:** 2014-04-29

**Authors:** Ulfah Rimayanti, Yoshiaki Kiuchi, Shohei Uemura, Joji Takenaka, Hideki Mochizuki, Makoto Kaneko

**Affiliations:** 1 Department of Ophthalmology and Visual Science, Graduate School of Biomedical Sciences, Hiroshima University, Hiroshima, Japan; 2 Department of Mechanical Engineering, Graduate School of Engineering, Osaka University, Osaka, Japan; University of Missouri-Columbia, United States of America

## Abstract

**Purpose:**

To evaluate the displacement of the central ocular surface during non-contact tonometry with and without soft contact lenses and determine the factors associated with the displacement of the central ocular surface and intraocular pressure (IOP) reading changes caused by wearing soft contact lenses (CLs).

**Methods:**

One eye each in 21 subjects was studied. The cornea was photographed using a high-speed camera at 5,000 frames/sec during non-contact tonometry without contact lenses (NCL), with -5.0 diopters (D), -0.5 D and +5.0 D CL. The displacement of the ocular surface and the factors affecting displacement at the IOP reading and maximum displacement time were investigated.

**Results:**

The IOP readings while wearing +5 D CL were significantly higher than those obtained while wearing -5 D CL. The ocular surface displacement between +5 D CL and other groups were significantly different. A significant positive correlation was found between the ocular surface displacement of subjects at the IOP reading time and the IOP obtained with the non-contact tonometer. A significant negative correlation was found between the ocular surface curvature and the IOP obtained using the non-contact tonometer. The radius of curvature of the ocular surface affected the displacement during the IOP reading and maximum displacement time.

**Conclusions:**

Our results indicate that soft contact lens use changes the ocular surface behavior and IOP readings during non-contact tonometry. The radius of curvature of the eye affects the ocular surface displacement and IOP readings in this situation.

## Introduction

Obtaining accurate measurements of the intraocular pressure (IOP) is an important part of ocular examinations. The use of non-contact tonometry for IOP measurement is now well established [1], [2]. Non-contact tonometry employs an applanation method using a standardized air puff to flatten the cornea [3]. As the pressure of the air puff increases to deform the cornea, the corneal surface behaves like a mirror and reflects light to the detector on the tonometer. The detected amount of reflected light reaches a maximum when the cornea is approximately flat, and the instrument records the time required to flatten the cornea and displays the IOP corresponding to that duration [4]-[6].

Soft contact lenses are commonly used to correct refractive errors of the eye and protect the ocular surface from ocular damage and after corneal surgery. Monitoring the IOP is an essential part of daily clinic activity, and tonometry through the soft contact lenses is convenient especially when treating subjects with ocular surface diseases. The effects of conventional soft contact lenses of low power on the IOP have been reported to be negligible [7]–[9]. Nevertheless, other studies have found that the IOP measured on non-contact tonometry through soft contact lenses is altered and that the changes depend on the lens power, curvature, thickness and rigidity [10]–[15]. Most reports have examined the correlations between IOP readings and lens factors, and there is no agreement as to why IOP readings change after wearing soft contact lenses.

Corneal stiffness is a general term that describes the resistance of the cornea to deformation and is the primary factor influencing the ability to measure the IOP [16], [17]. Kempf et al. developed a new method to evaluate corneal stiffness noninvasively by observing the degree of corneal displacement during non-contact tonometry using a high-speed camera [3], [4]. They proposed the use of non-contact stiffness measurements to evaluate the behaviour of the human eye during the application of dynamic forces and reported that the degree of corneal displacement during tonometry is one indicator of corneal stiffness [3], [4].

In order to understand why IOP readings are changed by soft contact lens use, we employed the methods developed by Kempf et al. [3], [4], and evaluated the extent of central corneal displacement at a designated time following an air puff application from a non-contact tonometer. We compared the degree of ocular surface movement in subjects not wearing contact lenses (NCL) to that observed in subjects wearing soft hydrogel contact lenses (CLs). The power of the lenses was −5.0 diopters (D), −0.5 D and +5.0 D (CL −5.0 D, CL −0.5 D and CL +5.0 D). We also investigated factors that were significantly associated with the difference in the extent of ocular surface displacement. We found that contact lens use changed IOP readings and the amount of ocular surface displacement. Our findings indicate that the radius of curvature of the anterior surface of the eye is associated with the amount of ocular surface displacement and IOP readings obtained with the non-contact tonometer.

## Subjects and Methods

### Subjects

The subjects consisted of 21 healthy young volunteers between August and December 2009. This study was approved by the Institutional Review Board of Hiroshima University, Japan. All study procedures adhered to the tenets of the Declaration of Helsinki. Written informed consent was obtained from each subject after informing them of the nature and possible complications of the examination procedures. Subjects with a history of ocular surgery or any type of eye disease other than refractive errors were excluded.

All subjects underwent standard ophthalmological examinations, including measurement of the best-corrected visual acuity, slit-lamp examinations and ophthalmoscopy. The corneal central thickness (CCT) was measured with a specular microscope (SP-2000p; Topcon Corporation, Tokyo, Japan). The refractive error (spherical equivalent) and corneal curvature were measured with an autorefractor/autokeratometer (ARK-700A; NIDEK Co.,Ltd., Gamagori, Japan).

Before beginning the measurements, we confirmed that the non-contact tonometer (CT-90A; Topcon Corporation, Tokyo, Japan) would apply an air puff to the cornea with the same pressure distribution, as previously described in detail [3]. The measurement of IOP in each subject using the non-contact tonometer was initially obtained without the use of contact lenses. Then, the IOP was measured while the subject wore hydrogel ACUVUE Contact Lenses (Johnson & Johnson K.K. Vision Care, Tokyo, Japan) of −5.0 D, −0.5 D and +5.0 D, 8.7 mm base curve, 14.0 mm diameter, with 58% water content. Thicknesses of contact lenses are 0.084 mm for −5.0 D, 0.124 mm for −0.5 D, and 0.21 mm for +5 D. The curvature of the contact lens surface is not open to the public (Comment from Johnson & Johnson K.K. Vision Care, Tokyo, Japan).

During tonometry, the cornea was photographed in profile using a high-speed camera oriented perpendicular to the geometrical axis of the eye. Fifteen minutes after photographing the cornea, we measured the IOP using Goldmann applanation tonometry.

### High-speed photography

A high-speed digital camera (Memrecam GX-1; Nac Image Technology Inc, Tokyo, Japan) with a field of 1,280 x 1,024 pixels was used to photograph the corneal profile at 5,000 frames/s (1 frame/0.2 ms). The cornea was illuminated by two infrared light sources to assure that high quality optical images would be obtained at this high frame rate. The high-speed camera shutter was synchronized to the non-contact tonometer switch, and the photographic system was designed to allow the camera to photograph the cornea from the onset of the air puff for 30.0 ms.

In order to examine the corneal surface in detail, the photographed images were processed to obtain a binary format of the image using the Microsoft Visual Studio 2008 (Microsoft, USA) and OpenCV 1.0 (Intel Corporation, USA) software programs. With the edge of the image defined, the surface of the image of the cornea was fitted to circles using the least squares method. This made it possible to find the center coordinate of the circle on the image and the radius of the ocular surface curvature. The coordinate at the top of the corneal edge was defined as the point of intersection of the corneal edge with the geometric x-axis extending from the center coordinate of the ocular surface image. The amount of displacement of the corneal image from the initial position was computed from the edge of the cornea frame-by-frame [3], [4]. The extent of displacement was obtained in each subject without wearing contact lenses, then while wearing contact lenses of −5.0 D, −0.5 D and +5.0 D. We used the term ocular surface displacement in this study. The ocular surface describes the outermost part of the eye, with and without contact lens wear.

### Determination of the ocular surface displacement

The time course of the changes in corneal shape following application of the air puff can be separated into several phases: the time when the cornea starts to bend inward, the time when the cornea becomes flat (IOP reading time), the time of maximum deformation when the cornea is concave and the recovery phase when the cornea returns to its original form. During the recovery phase, the cornea becomes flat for a second time. The initial flattening time has been shown to be strongly correlated with the IOP [5].

### Statistical analyses

The significance of the differences in the IOP readings and ocular surface displacement between the eyes treated without contact lenses and the eyes treated with −5.0 D, −0.5 D and +5.0 D contact lenses was determined using the Kruskal-Wallis nonparametric test followed by the Steel-Dwass multiple comparison test. Spearman's correlation analysis was used to determine the relationships between the amount of corneal displacement at the IOP reading time and the IOP obtained by non-contact tonometer, the radius ocular curvature and IOP obtained using the non-contact tonometer. The relationships between the IOP readings obtained by non-contact tonometry, amount of ocular surface displacements at IOP reading time and maximum displacement and the age, gender, CCT, radius of curvature of the ocular surface, spherical equivalent and IOP obtained with the Goldmann applanation tonometer were analyzed using stepwise and multiple linear regression analyses. A *P* value of <0.05 was considered to be statistically significant. The statistical analyses were performed using the JMP 10.0 version software program (SAS Institute Inc. Cary, NC).

## Results

### Demographics of the subjects

The subjects included 13 females and eight males, with a mean age of 22.1+1.6 years. The average central corneal thickness and refractive error are presented in Table 1.

**Table 1:** The values are presented as the means ± standard deviations.

Parameter	Average	Range
Age, y	22.14±1.56	20–27
Gender, W/M	13/8	-
CCT, µm	536.19±29.83	484–582
Spherical equivalent, D	−4.24±2.64	−8.8–0

W/M, women/men.

### IOP readings and radius of curvature of the ocular surface

The IOP values measured using Goldmann applanation tonometer and the non-contact tonometer were not significantly different in the subjects treated without contact lenses (*P* = 0.194). The IOP values measured using the non-contact tonometer and the radius of curvature of the ocular surface obtained without contact lenses and with contact lenses of −5.0 D, −0.5 D and +5.0 D are shown in Table 2. The IOP values of the eyes treated with CL +5.0 D were highest, followed by those observed in the eyes treated without contact lenses and those treated with CL −0.5 D and CL −5.0 D. The differences in IOP were not significant between any two groups, except for the comparison of the eyes treated with contact lenses of −5.0 D and +5.0 D (*P* = 0.012).

**Table 2:** Intraocular pressure measured using a non-contact tonometer and the radius of ocular surface curvature obtained with a high-speed camera.

Parameter	CL −5 D	CL −0.5 D	NCL	CL +5 D
IOP (mmHg)	13.26±1.78*	14.01±2.32	14.73±2.41	15.52±2.25*
Radius of ocular curvature (mm)	8.3±0.86* † §	7.59±0.73§	7.52±0.58† ‡	6.94±0.6* ‡

Significant differences were determined according to a post-hoc analysis.

*Significant difference for CL −5 D and CL +5 D with respect to the IOP measured according to non-contact tonometer (*P* = 0.012) and the radius of ocular curvature (*P*<0.0001).

† Significant difference for NCL and CL −5 D in the radius of ocular curvature (*P* = 0.012).

‡ Significant difference for NCL and CL +5 D in the radius of ocular curvature (*P* = 0.042).

§ Significant difference for CL −5 D and CL −0.5 D in the radius of ocular curvature (*P* = 0.049).

The radius of curvature of the cornea along the vertical meridian obtained with a keratometer was 7.81±0.19 mm, while that obtained with a high-speed camera was 7.52±0.58 mm. There were no significant differences between these two parameters (*P* = 0.157). There were significant differences between the radius of the ocular surface curvature obtained with the high-speed camera when the subjects were not wearing contact lenses and when the subjects were wearing CL of −5.0 D (*P* = 0.012), and between NCL and CL of +5.0 D (*P* = 0.042), −5.0 D CL and −0.5 D CL (*P* = 0.049) and CL −5.0 D and CL +5.0 D (*P*<0.0001).

### Comparison of ocular surface displacement at the IOP reading time and the maximum displacement time

At the time of IOP measurement, only the difference between the −5.0 D CL and +5.0 D CL groups was significant (*P* = 0.003, Table 3). The amount of maximum displacement was significantly different between the NCL and +5.0 D CL groups (*P*<0.0001), the −5.0 D CL and −0.5 D CL groups (*P* = 0.043), the +5.0 D CL and −0.5 D CL groups (*P* = 0.0002) and the CL −5.0 D and CL +5.0 D groups (*P*<0.0001).

**Table 3:** Comparison of ocular surface displacement at 14.8

Ocular surface displacement	CL −5 D, µm	CL −0.5 D, µm	NCL, µm	CL +5 D, µm
IOP reading time	71.03±33.01*	98.77±38.65	100.14±39.76	131.02±51.62*
Maximum displacement time	314.60±55.59* ‡	367.25±50.27‡ §	359.85±44.87†	458.73±58.79* † §

*Significant difference for CL −5 D and CL +5 D at the IOP reading time (*P* = 0.003) and maximum displacement (*P*<0.0001).

† Significant difference for NCL and CL +5 D at maximum displacement (*P*<0.0001).

‡ Significant difference for CL −5 D and CL −0.5 D at maximum displacement (*P* = 0.043).

§ Significant difference for CL +5 D and CL −0.5 D at maximum displacement (*P* = 0.0002).

**Figure 1:**
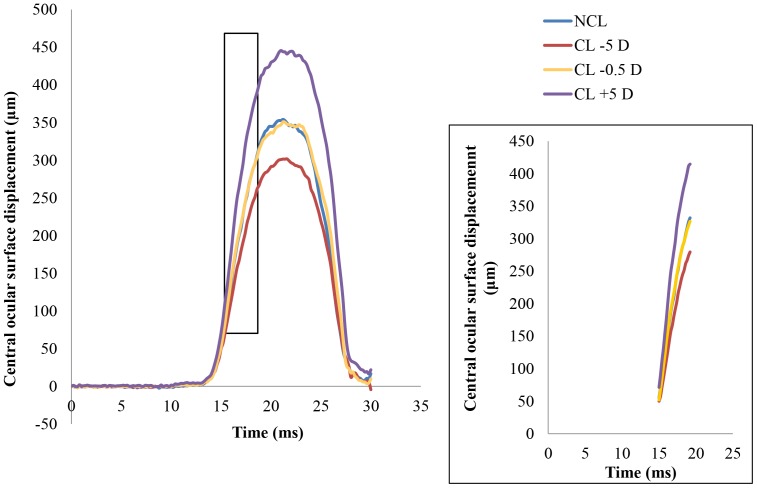
The average of central ocular surface displacement of subjects. Contact lens use changes the amount of ocular surface displacement.

The average ocular surface displacement profile during non-contact tonometry is shown in Figure 1. The average ocular surface displacement while wearing −5.0 D CL was smaller than that observed without wearing CL. The average displacement while wearing +5.0 D CL was larger than that observed without wearing CL. The difference in ocular surface displacement between the subjects wearing −0.5 D CL and NCL was not significant.

### Relationship between ocular surface displacement at the time of IOP reading and IOP reading obtained with the non-contact tonometer

**Figure 2:**
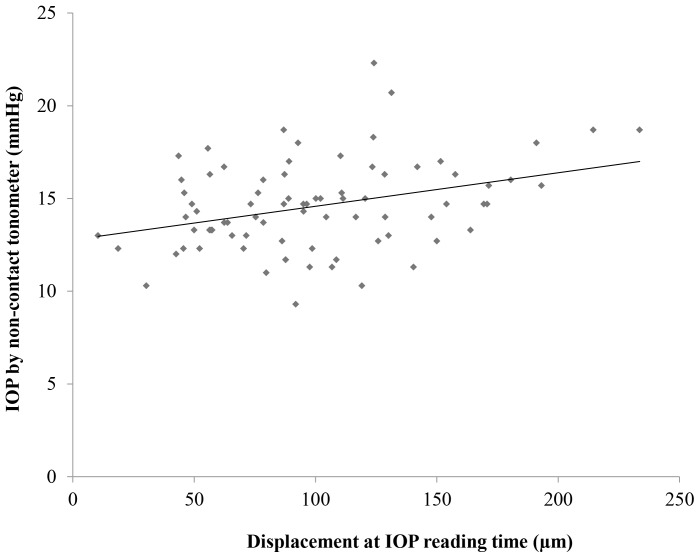
The relationship between the ocular surface displacement at the IOP reading time and IOP. The amount of ocular surface displacement at the IOP reading time showed a significant positive correlation with the IOP obtained using the non-contact tonometer (R^2^ = 0.1187, *P* = 0.007).

Scatter plots with a regression line between the amount of ocular surface displacement at the IOP reading time and the IOP obtained by the non-contact tonometer is presented in figure 2. A significant positive correlation was found between the ocular surface displacement of subjects and the IOP obtained by the non-contact tonometer. The bigger the ocular surface displacement, the higher the IOP (R^2^ = 0.1187, *P* = 0.007).

### Relationship between the ocular surface curvature and IOP reading obtained with the non-contact tonometer

**Figure 3:**
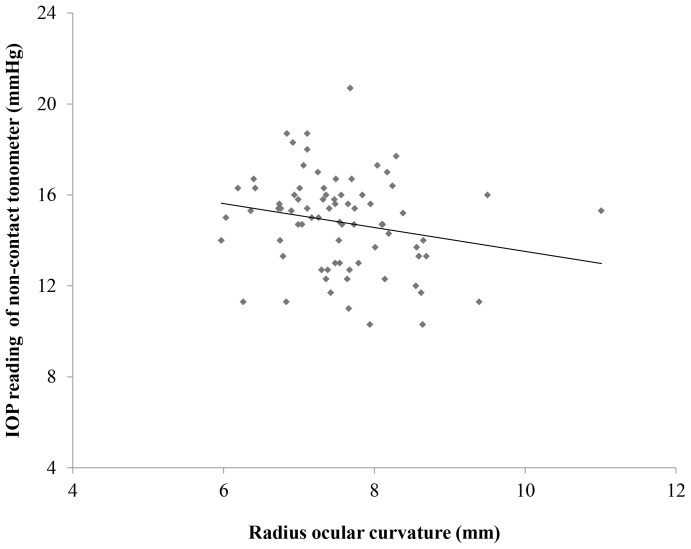
The relationship between the radius ocular surface and IOP. The radius ocular curvature showed a significant negative correlation with the IOP obtained using the non-contact tonometer (R^2^ = 0.0443, *P* = 0.028).

A scatter plot showing the relationship between the ocular surface curvature and the IOP obtained using the non-contact tonometer is presented in Figure 3. A significant negative correlation was found between the ocular surface curvature and the IOP obtained with the non-contact tonometer (R^2^ = 0.0443, *P* = 0.028).

### Independent predictors of IOP readings obtained with the non-contact tonometer and the average ocular surface displacement at the time of IOP reading and maximal displacement

The results of the multiple linear regression analyses with selected variables are shown in Tables 4, 5, and 6. As shown in table 4, among age, gender, CCT, radius of curvature of the ocular surface, refractive error, IOP obtained with the Goldmann applanation tonometer, the ocular surface displacement at the time of IOP reading and maximal ocular surface displacement, the multiple linear regression analyses showed that IOP obtained with the Goldmann applanation tonometer, spherical equivalent and the ocular surface displacement at the time of IOP reading were found to be predictive factors for the IOP readings obtained using non-contact tonometry (*P*<0.0001, *P* = 0.048 and *P* = 0.035, respectively). For the ocular surface displacement at the peak and maximum displacement times, the multiple linear regression analyses showed the radius of the ocular surface curvature (*P* = 0.002 and *P*<0.0001 respectively) to be a predictive factor for ocular surface displacement (Tables 5 and 6). At the time of IOP reading, an increase in the radius of ocular surface curvature of as much as 1 mm was associated with a mean decrease of 19.008 µm in ocular surface displacement (*P* = 0.002).

**Table 4:** Results of the multiple linear regression analysis with the stepwise method in which the IOP obtained by non-contact tonometry was the outcome variable.

Parameter	Coefficient	Standard Error	*P* Value
IOP obtained by Goldmann applanation tonometer	0.48	0.081	<0.0001
Spherical equivalent	−0.174	0.086	0.048
Displacement at IOP reading time	0.013	0.006	0.035
Maximum displacement	−0.008	0.004	0.059
Intercept	0.777	6.883	0.911

Predictors were selected from CCT, age, gender, SE, radius of ocular curvature, IOP obtained by Goldmann applanation tonometer, the ocular surface displacement at IOP reading time and the maximal ocular surface displacement time.

**Table 5:** Results of the multiple linear regression analysis with the stepwise method in which the average ocular surface displacement at the time of IOP reading was the outcome variable.

Parameter	Coefficient	Standard Error	*P* Value
Radius of ocular surface curvature	−19.008	5.815	0.002
Age	6.426	3.705	0.087
Intercept	105.079	88.076	0.237

Predictors were selected from among CCT, age, gender, SE, the radius of ocular curvature and the IOP obtained using the Goldmann applanation tonometer.

**Table 6:** Results of the multiple linear regression analysis with the stepwise method in which the maximal ocular surface displacement was the outcome variable.

Parameter	Coefficient	Standard Error	*P* Value
Radius of ocular surface curvature	−43.743	8.479	<0.0001
Spherical equivalent	−5.117	2.752	0.067
Intercept	684.224	66.701	<0.0001

## Discussion

Our results which showed that IOP of naked eyes obtained by Goldmann applanation tonometry was similar to IOP obtained by non-contact tonometry are in agreement with previous studies [18], [19]. We found a tendency toward higher IOP readings associated with the use of positive thicker contact lenses and lower IOP readings associated with the use of negative thinner contact lenses. Our results showed that the average IOP in the subjects wearing −5.0 D CL was significantly different from that observed in the subjects wearing +5.0 D CL. Our findings confirm the results of other studies showing that changes in IOP depend on the lens power [11]–[15]. The IOP readings increased in association with hyperopic thicker lens use [11]–[13], while the IOP values in the subjects wearing myopic thinner lenses tended to be underestimated [14], [15].

Young's modulus of the cornea was reported to be 0.29±0.06 Mpa [20], while that of hydrogel soft contact lenses is 0.29±0.03 Mpa (Information obtained from Johnson & Johnson K.K. Vision Care, Tokyo, Japan). Although Young's modulus of the cornea and that of contact lenses are similar, whether contact lenses and the cornea behave together remains to be verified. The thicker combined ocular surface does not explain the low IOP readings observed in eyes treated with −5.0 D contact lenses because, when wearing −5.0 D lenses, the subjects have a thicker ocular surface component than that observed in eyes treated without contact lenses. Our results suggest that corneas treated with soft contact lenses do not simply respond to the power from outside as a single thicker component. Contact lenses stay on the cornea due to the surface tension of the tear film [21]. A small external force, such as blinking, can easily cause the lens to move. The thickness of the tear layer is 0.007–0.008 mm [22]. Our camera did not have the ability to capture tear film movement during tonometry or analyze the behavior of the cornea and soft contact lens separately.

We also found that eyes wearing +5.0 D CL had the largest amount of displacement of the central ocular surface and highest IOP readings, while eyes wearing −5.0 D CL had the smallest amount of displacement and lowest IOP readings. In contrast, Kiuchi et al. [16] and Nakakura et al. [17] found that a lower IOP is associated with a greater degree of displacement of the cornea. The multiple linear regression analyses used in earlier reports selected the IOP as the strongest factor affecting the extent of corneal displacement. Under such conditions, the corneas with lower IOP readings exhibited greater displacement. This is a large discrepancy between the findings of previous studies and our results. Mark reported a positive correlation between corneal curvature and IOP readings: a flatter corneal curvature was found to be associated with lower tonometric readings [10]. Our multiple regression analysis found that the radius of curvature of the ocular surface strongly affected the amount of central ocular surface displacement in the eyes treated with soft contact lenses during the IOP reading time and maximum displacement time.

Our statistical analysis using multiple linear regression showed that the Goldmann IOP reading for the naked eye, spherical equivalent and the ocular surface displacement at the time of IOP reading were the predictive factors for the IOP readings by noncontact tonometer through the contact lens. The Goldmann IOP reading is known to be well correlated with the IOP reading obtained with non-contact tonometers [18], [19]. The effects of soft contact lenses of low power on the IOP are small [7]–[9]. Therefore, it is reasonable that the Goldmann IOP reading was selected as a significant factor predicting the IOP values over the soft contact lenses obtained using non-contact tonometers. The spherical equivalent was also selected as a factor determining the IOP readings obtained with the non-contact tonometer over soft contact lens. However, the spherical equivalent does not explain differences between the IOP readings obtained while wearing +5.0 D and −5.0 D soft contact lenses. Previous studies using non-contact tonometers have found the spherical equivalent to correlate with CCT [23], but not IOP [24], [25].

The ocular surface curvature was identified to be a strong factor affecting the degree of ocular surface displacement during the IOP readings and the maximum displacement time (Tables 5 and 6) by stepwise and multiple linear regression analyses. In addition, scatter plots showed a significant relationship between the IOP reading and the ocular surface curvature (Fig. 3). Our subjects with −5.0 D CL had a flatter ocular surface, and it might be easier to reach a flat condition. This is one potential reason why underestimation of the IOP associated with a small degree of displacement was observed. On the other hand, eyes treated with +5.0 D contact lenses have a steeper surface and require a longer time to reach a flat surface. This leads to the overestimation of IOP and a larger amount of displacement of the ocular surface. We analyzed the amount of ocular surface displacement at the time of IOP reading and maximum displacement, as well as age, gender, CCT, the radius of curvature of the ocular surface and the spherical equivalent and IOP obtained with the Goldmann applanation tonometer, as confounding factors for determining the IOP readings obtained with non-contact tonometry. Nevertheless, other factors, such as the surface tension between the cornea and the contact lens or the layer of tears with a buffering function, may have had some effects on the IOP readings obtained over the contact lenses. The present study system was unable to evaluate the effects of such factors on the IOP readings. This is one limitation of our study.

There are other limitations in this study. The number of subjects was small, and the reproducibility of the measurements was not high [16]. Recent studies of a new non-contact tonometer used in combination with an ultra-high-speed Scheimpflug camera have reported very good repeatability of ocular deformation parameters in healthy subjects and patients with ocular hypertension and glaucoma [26], [27]. This new instrument may allow clinicians to more easily obtain more reliable deformation values in a larger number of subjects.

Aging affects the extent and shape of the displaced cornea [3], [16], [17]. In this study, all of the subjects were young, and the amount of corneal displacement in younger individuals gradually decreases after maximum displacement until the cornea reaches the initial position. The profile of corneal deformation over time is bell-shaped (Figure 1), while that for elderly individuals differs because, after corneal deformation, the corneal shaped remains fixed for a few milliseconds, then starts to return until the cornea reaches its original shape and position [3], [28]. A line graph of the changes in the extent of central corneal deformation is bell-shaped with a shoulder bump at the late phase in older subjects [3], [28]. The effects of contact lens use on corneal bioproperties are different from those caused by aging. Contact lens use changes the amount, not the shape, of deformation.

In conclusion, we found that wearing contact lenses during non-contact tonometry altered the ocular surface behaviour and IOP readings. Our findings indicate that the radius of ocular surface curvature is associated with the changes of ocular surface displacement and IOP readings in this situation.
