# Correction: Ocular Surface Displacement with and without Contact Lenses during Non-Contact Tonometry

**DOI:** 10.1371/journal.pone.0104308

**Published:** 2014-07-28

**Authors:** The PLOS ONE Staff

The legend for Table 3 incorrectly lists the measurement of 14.8ms. The correct legend is below.

## Table 3. Comparison of ocular surface displacement at the IOP reading time and the maximum displacement time.
